# Increased Susceptibility of the CD57^−^ NK Cells Expressing KIR2DL2/3 and NKG2C to iCasp9 Gene Retroviral Transduction and the Relationships with Proliferative Potential, Activation Degree, and Death Induction Response

**DOI:** 10.3390/ijms222413326

**Published:** 2021-12-11

**Authors:** Anastasia I. Palamarchuk, Nadezhda A. Alekseeva, Maria A. Streltsova, Maria O. Ustiuzhanina, Polina A. Kobyzeva, Sofya A. Kust, Maria V. Grechikhina, Anna A. Boyko, Olga A. Shustova, Alexander M. Sapozhnikov, Elena I. Kovalenko

**Affiliations:** 1 Shemyakin and Ovchinnikov Institute of Bioorganic Chemistry, Russian Academy of Sciences, st. Miklukho-Maklaya, 16/10, 117997 Moscow, Russia; palanastasia@yandex.ru (A.I.P.); nadalex@inbox.ru (N.A.A.); mstreltsova@mail.ru (M.A.S.); mashaust1397@gmail.com (M.O.U.); polina-kobyzev@yandex.ru (P.A.K.); sonya.erokhina@gmail.com (S.A.K.); marygrec@mail.ru (M.V.G.); boyko_anna@mail.ru (A.A.B.); olga_shustova@list.ru (O.A.S.); amsap@mx.ibch.ru (A.M.S.); 2 Center of Life Sciences, Skolkovo Institute of Science and Technology, 121205 Moscow, Russia

**Keywords:** NK cells, genetic modifications, adoptive NK cells, HLA-DR, proliferation, retroviral vectors, iCasp9 gene, transduction, suicide switch

## Abstract

Nowadays, the use of genetically modified NK cells is a promising strategy for cancer immunotherapy. The additional insertion of genes capable of inducing cell suicide allows for the timely elimination of the modified NK cells. Different subsets of the heterogenic NK cell population may differ in proliferative potential, in susceptibility to genetic viral transduction, and to the subsequent induction of cell death. The CD57^−^NKG2C^+^ NK cells are of special interest as potential candidates for therapeutic usage due to their high proliferative potential and certain features of adaptive NK cells. In this study, CD57^−^ NK cell subsets differing in KIR2DL2/3 and NKG2C expression were transduced with the iCasp9 suicide gene. The highest transduction efficacy was observed in the KIR2DL2/3^+^NKG2C^+^ NK cell subset, which demonstrated an increased proliferative potential with prolonged cultivation. The increased transduction efficiency of the cell cultures was associated with the higher expression level of the HLA-DR activation marker. Among the iCasp9-transduced subsets, KIR2DL2/3^+^ cells had the weakest response to the apoptosis induction by the chemical inductor of dimerization (CID). Thus, KIR2DL2/3^+^NKG2C^+^ NK cells showed an increased susceptibility to the iCasp9 retroviral transduction, which was associated with higher proliferative potential and activation status. However, the complete elimination of these cells with CID is impeded.

## 1. Introduction

Cellular therapy based on the use of genetically engineered immune cells with enhanced functionality, in particular T and NK cells, is a promising strategy for cancer treatment. The application of allogeneic T cells in adoptive therapy is not widespread because of drawbacks such as severe adverse side effects related to graft-versus-host disease (GVHD). At the same time, the procedure of modified autologous T cell production is very laborious [1]. It was shown in clinical trials that NK cells might be safer than T cells for immunotherapy since the application of NK cells is associated with lower risks of GVHD, cytokine storm syndrome, and neurotoxicity [2]. The technologies for obtaining immunotherapeutically suitable activated NK cells with genetic modifications have been widely developing recently [3].

Despite the significant advantages, the usage of NK cells in the clinic remains challenging because of the low proliferative potential and minimal expansion of these cells accompanied by their resistance to genetic modifications. The efficiency of NK cell transduction by viral particles is usually much less in comparison with other cells of hematopoietic origin, which is caused by an enhanced antiviral protective system in NK cells [4,5].

Preliminary stimulation of NK cells by cytokines and feeder cells may increase the transduction efficiency, presumably by boosting their proliferative activity [6]. In this work, we used a promising method of NK cell activation with IL-2 and an irradiated K562-mbIL21 modified cell line that had been proposed earlier [7] and described in detail in our previous works [8,9]. However, even after the effective stimulation, only a part of the NK cells was susceptible to the genetic viral transduction [6]. In part, this phenomenon could be associated with a significant phenotypic and functional heterogeneity of NK cells represented in the peripheral blood [10]. Different NK cell subpopulations vary in their proliferative potential, and the lowest proliferative activity was found for the highly differentiated CD57^+^ subset [8,9,11].

Unlike T cells, NK cells express a diverse pool of various activating receptors, which can mediate a cytotoxic response against cancer cells even after the loss of transgene expression and potentiate the transduction of activating signals inside the cell. Due to the expression of CD16, which is an Fc receptor mainly exposed at the later differentiation stages, the NK cells are capable of antibody-dependent cellular cytotoxicity (ADCC). Such features make NK cells, especially those that have been genetically modified, rather efficient immunotherapeutic agents [12]. Moreover, in the process of differentiation, NK cells undergo a “licensing” procedure. NK cells possessing an inhibitory killer-cell immunoglobulin-like receptor (KIRs) “acquire a license” to perform cytotoxic functions. This process is possible due to the recognition of the corresponding HLA-I complex by an inhibitory KIR and the subsequent transduction of the inhibitory signal through the inner domain of the receptor [13]. Thus, applying the NK cell subpopulations which have undergone the “licensing” stage may increase NK cell immunotherapeutic anti-cancer effectiveness.

Similar to the adaptive immune cells, the repertoire of NK cells is shaped not only by the signals from the surrounding cells and soluble factors, but also by pathogens invading an organism. The human cytomegalovirus (HCMV) occupies a special place among these pathogens [14]. HCMV infection is associated with an increased proportion of NKG2C^+^ NK cells, part of which forms a pool of so-called adaptive NK cells. These cells are characterized by increased longevity, functional activity, and a more effective and faster response to repeated stimulation compared to conventional NK cells. Adaptive NK cells, in addition to the NKG2C expression, have high levels of HLA-DR and KIRs [15,16]. These NK cells are characterized by the increased expression level of CD57, a marker that is usually expressed on the surface of highly differentiated cells with low proliferative potential [11]. Previously, this molecule was considered to be a marker of terminal differentiation, but it was shown that the loss of CD57 expression by NK cells is still possible [8]. Recently, we have demonstrated that the less differentiated CD56^dim^CD57^−^NKG2C^+^ cells share some properties with the adaptive CD57^+^NKG2C^+^ cells and possibly represent their precursors [17]. Along with the higher proliferative potential compared to the CD57^+^NKG2C^+^ subpopulation, the CD56^dim^CD57^−^NKG2C^+^ subset possessed an increased functional activity characteristic of adaptive NK cells.

Numerous side effects of cell-based immunotherapy indicate the need for control over modified cells after infusion. Although the therapeutic application of NK cells is much safer than that of T cells, strict control over the modified cells before and after the infusion is required to manage the side effects. For example, retroviral transduction can result in insertional mutagenesis as well as the unintentional targeting of immune cells and healthy tissues [3]. In addition, enhancing the NK cell functional activity through genetic modification can promote cell hyperactivation after the infusion, which increases the risk of side effects.

The insertion of the genes capable of inducing cell suicide, separately or simultaneously with other genetic modifications, can provide a valuable “safety switch off” for autonomous, inadequately functioning infused cells. Thus, the use of such constructions allows for the prevention or stops the development of severe side effects in advance [18]. To date, several suicide switches for modified cells have been developed, but the absence of side effects has only been reported for the caspase-9-based constructs [19]. The iCasp9 suicide construct consists of two parts: the FK506-F36V domain binding to a dimerizer (AP20187 or AP1903), and the caspase 9 site (Δcaspase9) without CARD (Caspase activating recruitment domain), connected by a Ser-Gly-Gly-Gly-Ser linker. ΔCaspase9 dimerization and activation followed by a chemical inducer of dimerization (CID) treatment triggers apoptosis in iCasp9-modified cells [20]. For example, for iCasp9-NK-92 cells, incubation in the medium with 10 nM CID for 30 min was sufficient to activate iCasp9 in all cells, and an incubation of 26 h to decrease the proportion of living cells to less than 2% [21]. The advantages of the iCasp9 suicide construct include the low immunogenicity of its components, its function as a non-toxic activating agent, and its high rate of apoptosis induction.

In this study, we analyzed the effectiveness of retroviral transduction with the iCasp9 gene-containing construct of pre-activated subpopulations of less differentiated, proliferatively active CD57^−^NK cells that possess distinct expression patterns of the KIR2DL2/3 and NKG2C receptors. Proliferative activity, expression of the activation marker HLA-DR and the maturation marker CD57, and susceptibility to apoptosis induced by the CID treatment were compared between different iCasp9-transduced NK cell subpopulations obtained from HCMV seropositive healthy individuals.

## 2. Results

### 2.1. NK Cells with Surface KIR2DL2/3 Expression Show Greater Retroviral Transduction Efficiency Compared to KIR2DL2/3-Negative NK Cells

Since low transduction efficiency had been shown for CD57^+^ NK cells [6], our current efforts were focused on discovering a CD57^−^ NK subpopulation that would be characterized by high susceptibility to retroviral transduction and possess optimal functional characteristics and high proliferative potential.

**Figure 1:**
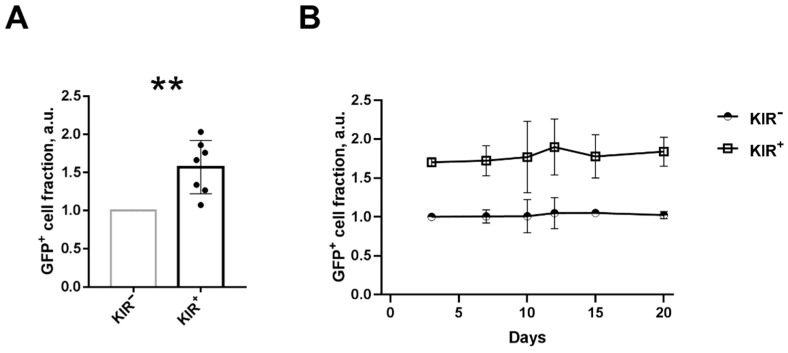
The transduction of NK cell subsets differing in KIR2DL2/3 expression. (**A**) The GFP^+^ cell fraction in sorted KIR2DL2/3^+^ and KIR2DL2/3^−^ NK cell subsets, 5 days after the transduction. The averaged data from different donors (*n* = 8) are presented. (**B**) Dynamics of the GFP^+^ cell fraction in KIR2DL2/3^+^ and KIR2DL2/3^−^ NK cell subsets after the transduction (*n* = 2). The GFP+ cell fraction was measured in arbitrary units (a.u.) as the GFP level in the KIR2DL2/3^+^ subset divided by the GFP level in the KIR2DL2/3^−^ subset for each donor. In this and the following figures, KIR2DL2/3 is designated as KIR. Statistical analysis was performed using the paired *t*-test (** *p* < 0.01); means ± SE are shown.

We have previously shown that NK cells modified by gamma-retroviral particles comprising the *GFP-P2A-hTERT* gene contained a greater fraction of KIR2DL2/3^+^ cells one week after transduction [22]. On the one hand, this could have resulted from the higher proliferative rate of KIR2DL2/3^+^ NK cells after the transduction procedure. On the other hand, it might be indicative of a greater susceptibility of KIR2DL2/3^+^ NK cells to transduction. First, we decided to test whether the fraction of KIR2DL2/3^+^ NK cells possesses traits that increase the efficiency of transduction. For this purpose, freshly isolated CD57^−^ NK cells were sorted according to KIR2DL2/3 expression into KIR2DL2/3^+^ and KIR2DL2/3^−^ subsets, then stimulated for 7 days with IL-2 and K562-mbIL21, and transduced with retroviral particles bearing the GFP reporter protein in a 96-well plate. In most of the experiments, the highest transduction efficiency was observed in the KIR2DL2/3^+^ NK cell subset rather than in the KIR2DL2/3^−^ subset (Figure 1A). Thus, there is a notable association between NK cell transduction efficiency and the surface expression of the KIR2DL2/3 receptors. Alterations in the size of the GFP^+^ fraction in the NK cell subsets have been traced to three weeks after the transduction. No increase in the proportion of the GFP^+^ fraction in the KIR2DL2/3^+^NK subset was observed during cultivation (Figure 1B), which confirms that KIR2DL2/3^+^ NK cells do not have any proliferative advantage. Thus, an association between the presence of KIR2DL2/DL3 receptors on the surface of NK cells and their greater susceptibility to the retroviral transduction was shown.

### 2.2. Frequencies of NK Cells Expressing KIR2DL2/3 and/or NKG2C Receptors Differ in CD56^bright^ and CD56^dim^CD57^−^ NK Cell Subsets

In our previous work, we detected a higher HLA-DR expression level indicating a greater activation state, and a higher content of KIR2DL2/3^+^ cells in the modified GFP^+^ NK cells compared to GFP-negative cells [22]. Other studies have shown that a higher level of HLA-DR expression is inherent in NKG2C^+^ NK cells, including adaptive NK cells associated with HCMV infection [17,23], which are also characterized by KIR expression . Both classical adaptive NKG2C^+^CD57^+^ NK cells and their potential precursors, NKG2C^+^CD57^−^ NK cells, possess specific functional features [17], which make them interesting for potential use in immunotherapy. Hence, we decided to perform a more precise investigation of the subpopulations of NK cells that differ in KIR2DL2/3 and NKG2C expression, particularly to compare their susceptibility to modification by retroviral transduction. Taking into account the higher resistance of CD57^+^ NK cells to retroviral transduction as previously shown by Streltsova et al. [6], we focused on less differentiated CD57^−^ subsets.

**Figure 2:**
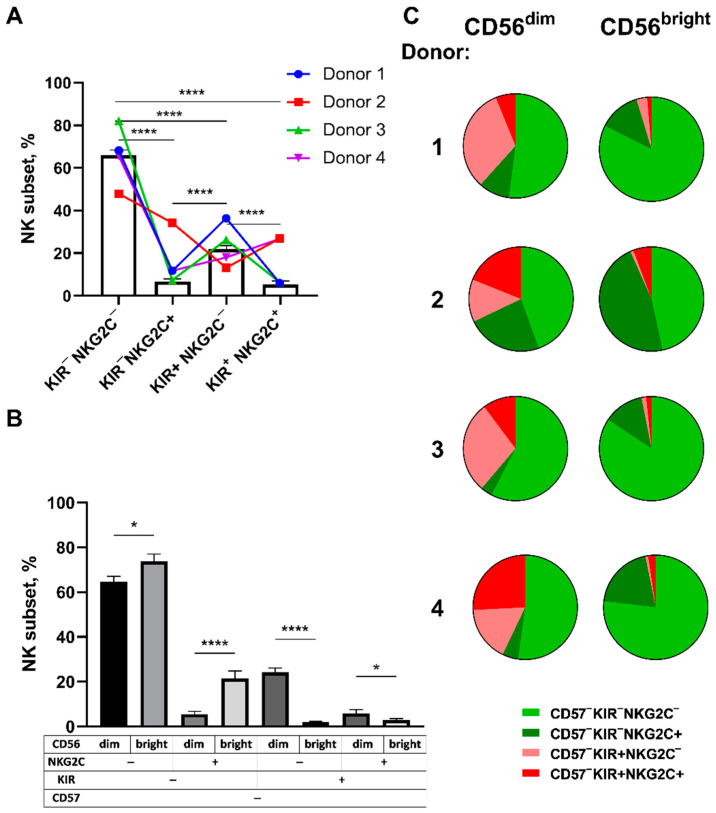
Comparison of ex vivo proportions of the CD57^−^KIR2DL2/3^+/−^ NKG2C^+/−^ NK cell subsets in HCMV^+^ healthy donors. (**A**) Proportions of the KIR2DL2/3^+/−^ NKG2C^+/−^ subsets in CD57-negative NK cells from different donors, n=16 (bars, means ± SE). The four donors selected for transduction experiments are highlighted with colors and lines. (**B**) The averaged KIR2DL2/3^+/−^NKG2C^+/−^ NK cell proportions in the CD56^dim^CD57^−^ and CD56^bright^CD57^−^ subpopulations, *n*=16 (means ± SE). (**C**) The proportions of CD57^−^KIR2DL2/3^+/−^NKG2C^+/−^ NK cells in the CD56^dim^ and CD56^bright^ subpopulations for each of the four individual donors selected for transduction experiments. Statistical analysis was performed using a paired *t*-test (* *p* < 0.05; **** *p* < 0.0001).

The distribution of the KIR2DL2/3- and NKG2C-expressing cells in the CD57^−^ fraction was studied in NK cells isolated ex vivo by magnetic separation from 16 healthy volunteers that were seropositive for HCMV (Figure 2A). The proportion of the KIR2DL2/3^+^NKG2C^+/−^ subsets in the selected fraction comprised about 30%. The KIR2DL2/3^+^NKG2C^+^ subset had the lowest frequency among the CD56^+^CD57^−^ NK cells (6% on average). The frequency of the KIR2DL2/3^−^NKG2C^+^ subset was also rather low, ranging from 3 to 20% of all CD56^+^CD57^−^ NK cells. The KIR2DL2/3^−^NKG2C^−^ subset was widely represented in the CD56^+^CD57^−^ NK cell fraction, with an average rate of 60%. The distribution of the KIR2DL2/3^+^ NKG2C^+/−^ subsets differed in the subpopulations of less mature CD56^bright^CD57^−^ and more mature CD56^dim^CD57^−^ NK cells (Figure 2B,C). A higher content of KIR2DL2/3-positive cells was observed among the CD56^dim^ NK cells in comparison with the CD56^bright^ NK cells (Figure 2B). At the same time, a large proportion of the KIR2DL2/3^−^ cells was observed in the CD56^bright^ cells as compared to the CD56^dim^ subset (Figure 2B,C).

NK cells from four HCMV-seropositive healthy volunteers were sorted into CD57^−^KIR2DL2/3^+/−^NKG2C^+/−^ cell subsets for subsequent analysis of retroviral transduction susceptibility. The proportions of the respective subsets ex vivo for each donor are presented in Figure 2C.

### 2.3. Transduction Efficiency Depends on KIR2DL2/3 and NKG2C Expression with the Higher Transduction Rate among the KIR2DL2/3^+^ and NKG2C^+^ NK Cells

For transduction experiments, four HCMV-seropositive volunteers with a high content of NKG2C^+^ cells (more than 13% of all NK cells) were selected. The CD57^−^KIR2DL2/3^+/−^NKG2C^+/−^ cell fractions were sorted from freshly isolated NK cells; sorting purity is shown in Figure S1. All subsets were stimulated with IL-2 and K562-mbIL21 feeder cells for seven days prior to the transduction.

To study the differences in transduction efficiency of the NK cell subsets, the γ-retroviral vector bearing the pMSCV-F-del Casp9.IRES.GFP construction was selected. The iCasp9 gene incorporation allows for the direct elimination of the modified NK cells after transplantation in order to prevent adverse effects. We prepared the common viral stock for all experiments to provide equal conditions in each transduction procedure. The number of transduction units (TU) per ml was tested on the NK-92 cell line, and the average number was 42,000 TU/ml. Viral aliquots were concentrated to reach 1.5 TU per one NK cell. The incorporation of the iCasp9 gene into the genome was qualitatively verified by the GFP fluorescence level.

**Figure 3:**
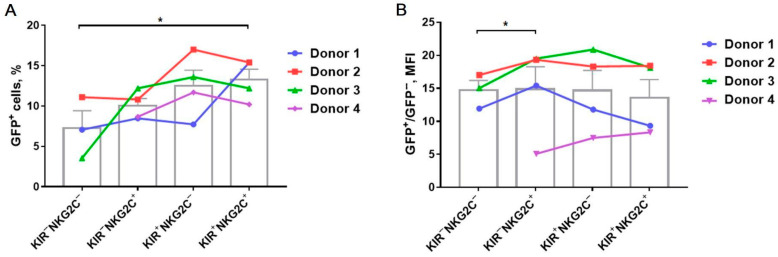
A comparison of the transduction efficiency of the CD57^−^KIR2DL2/DL3^+/−^NKG2C^+/−^ NK cell subsets stimulated with K562-mbIL21 and IL2 by retroviral particles with iCasp9 and *GFP* genes on the 5th day after infection. (**A**) The proportion of GFP^+^ NK cells in the indicated subsets. (**B**) The GFP mean fluorescence level (MFI) in the indicated NK cell subsets divided by the MFI of the respective GFP^−^ NK cells, *n* = 4. (**A**,**B**) Statistical analysis was performed using a paired *t*-test (* *p* < 0.05). Columns represent means ± SE. Data from individual donors are linked by lines.

All four analyzed NK cell subsets were susceptible to the retroviral transduction of the iCasp9-bearing construction to a certain extent, but the transduction efficiency varied among the subsets (Figure 3). A higher level of GFP^+^ NK cells was observed in the KIR2DL2/3-positive subsets (KIR2DL2/3^+^NKG2C^+^: 13.3 ± 1.3%, KIR2DL2/3^+^NKG2C^−^: 12.5 ± 1.9%) as compared to the KIR2DL2/3-negative subsets (KIR2DL2/3^−^NKG2C^+^: 10.0 ± 0.9%, KIR2DL2/3^−^NKG2C^−^: 5.5 ± 2.3%), with a significant difference between the double-positive and double-negative fractions (Figure 3A). The level of GFP fluorescence intensity was slightly higher in the KIR2DL2/3^−^NKG2C^+^ NK cell subpopulation, which may indicate a higher infection efficiency and susceptibility to transduction of part of the cells from this subset as compared to the KIR2DL2/3^−^NKG2C^−^ NK cell subset (Figure 3B).

### 2.4. The Proliferative Potential of the CD57^−^KIR2DL2/3^+^ Subsets Is Better Realized at Later Stages of Cultivation

One of the essential parameters for choosing NK cell subsets for therapeutic usage, apart from their functional characteristics, is their proliferative potential and the ability to generate a sufficient number of modified cells for the infusion. It was previously shown that the transduction efficiency of actively proliferating cells is higher than that of the slowly proliferating cells [8,9,11]. Thus, we compared the proliferative capacity of the CD57-negative NK cell subsets with different expression of the KIR2DL2/3 and NKG2C receptors.

**Figure 4:**
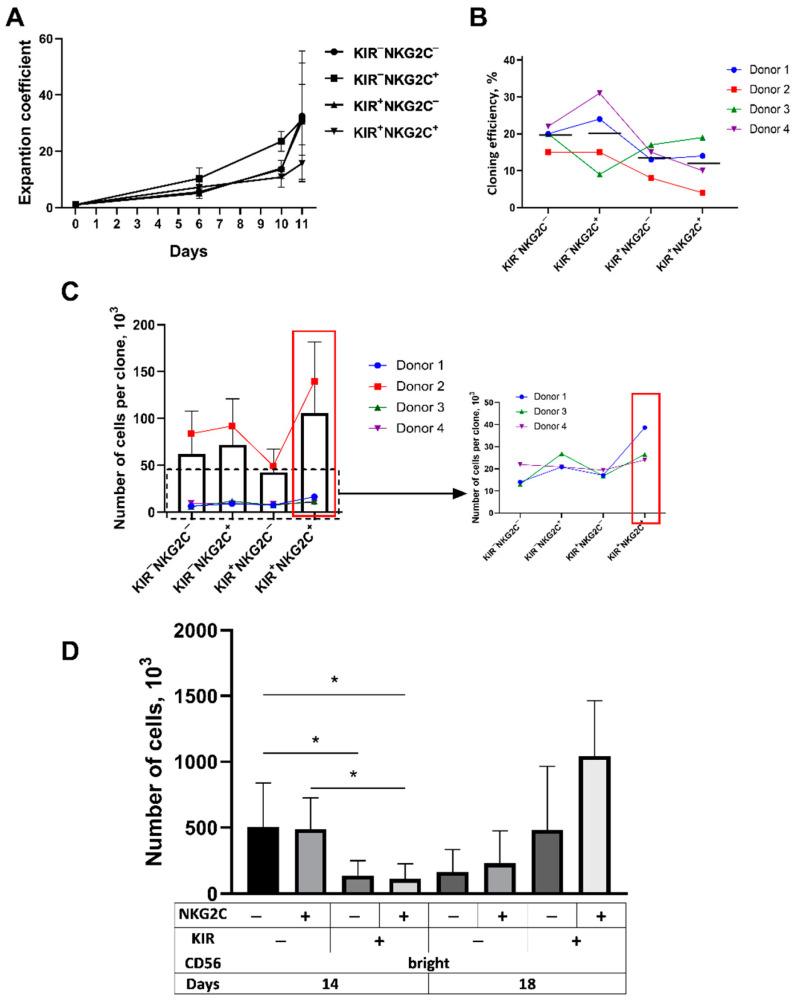
Proliferative activity of CD57^−^ NK cells differing in KIR2DL2/3 and NKG2C expression. (**A**) The expansion coefficient of the cultures obtained from CD57-negative KIR2DL2/3^+/−^ NKG2C^+/−^ NK cells from four different donors. (**B**) Cloning efficiency of CD57^−^KIR2DL2/3^+/−^NKG2C^+/−^ NK cells, measured after a 5-week stimulation with IL-2 and K562-mbIL21 feeder cells; black lines indicate means for four donors. The cloning efficiency was calculated as a percentage of the obtained clones from the total number of wells in which single NK cells were placed. (**C**) The average number of cells in a clone acquired from CD57-negative NK cell subsets after a 5-week stimulation with IL-2 and K562-mbIL21 cells, *n* = 4. (**D**) The average number of cells in CD56^bright^ subpopulations differing in the expression of KIR2DL2/3 and NKG2C on the 14th and 18th days of cultivation, *n* = 3. Statistical analysis was performed using a paired *t*-test (* *p* < 0.05); means ± SE are shown.

Cell number expansion was evaluated during the first 11 days after stimulation of the IL-2 and K562-mbIL21 cells in the CD57^−^KIR2DL2/3^+/−^NKG2C^+/−^ cell fractions. No statistical difference in the subset proliferation was shown. On day 10, the expansion of the most differentiated CD57^−^KIR2DL2/3^+^NKG2C^+^ subset was the smallest among the analyzed subpopulations (Figure 4A).

Additionally, the CD57^−^KIR2DL2/DL3^+/−^NKG2C^+/−^ NK cells of the four donors were cloned (progeny of the single cells were cultured) to compare the proliferative potential of NK cells from different fractions. A total of 120 cells from each subpopulation were sorted in a round-bottomed 96-well plate, with one cell per well according to a previously described technique [9]. Clonal cultures were cultured for five weeks. On average, less differentiated CD57^−^KIR2DL2/3^−^ NK cells formed more clones regardless of NKG2C expression, although no statistical difference was found between the groups (Figure 4B). Although the CD57^−^KIR2DL2/3^−^ NK cells produced more clones, the most abundant clonal cultures (with the largest number of cells per clone) in most cases were obtained from more differentiated CD57^−^KIR2DL2/3^+^NKG2C^+^ cells (Figure 4C). Possibly, in less differentiated subsets, the main contribution to the culture expansion is made by a large number of individual moderately proliferating cells, whereas cultures of more differentiated CD57^−^KIR2DL2/3^+^NKG2C^+/−^ subsets mainly expand due to a small number of very actively proliferating clones.

We assumed that certain cultures form more clones due to a higher amount of CD56^bright^ NK cells in them. The presence of CD25 on the surface of CD56^bright^ NK cells correlates with their better response to IL-2 stimulation, which can promote cell survival and proliferation [24]. Indeed, we observed that more than 93% of the CD56^bright^ NK cell subpopulation were CD57^−^KIR2DL2/3^−^ cells, whereas among the CD56^dim^ NK cell subset, these cells comprised no more than 70% (Figure 2C). Therefore, a greater number of CD56^bright^ cells in less differentiated CD57^−^KIR2DL2/3^−^ subsets compared to CD57^−^KIR2DL2/3^+^ subsets might lead to the increased cloning efficiency and expansion abilities of these subsets. In order to test this hypothesis, CD56^bright^ NK cell cultures that differed in the expression level of NKG2C and KIR2DL2/3 receptors were obtained. These cells were sorted by 100 cells per well of a 96-well plate. At initial cultivation stages, CD56^bright^ KIR2DL2/3^−^ NK cells proliferated more intensively as compared to more differentiated CD56^bright^KIR2DL2/3^+^ NK cells (Figure 4D); later, a more pronounced expansion was observed in the more differentiated and licensed CD56^bright^KIR2DL2/3^+^ cell fraction.

Thus, the increased expansion of CD57^−^KIR2DL2/3^−^ NK cell subsets at the early stages of cultivation was apparently related to a larger contribution of CD56^bright^ NK cells, which are less differentiated and more sensitive to IL-2 stimulation, although their proliferation does not seem to be long.

At the same time, the KIR2DL2/3^+^NKG2C^+^ subpopulation possesses a better proliferative potential in the long term due to the contribution of proliferating cells derived from larger clones with prolonged culturing. This could be one of the reasons for the higher susceptibility of this subset to the retroviral transduction.

### 2.5. Retroviral Transduction Success Is Associated with the Surface Level of the HLA-DR Activation Marker and CD57 De Novo Expression 

We studied the phenotypic features of the CD57^−^ NK cell cultures on the seventh day of stimulation with IL-2 and K562-mbIL21 cells before the transduction, and those of the modified and unmodified NK cells seven days after the transduction. At this time point, all cells damaged by the transduction procedure were already eliminated. Since the transduction efficiency was below 100%, each NK cell culture contained both effectively transduced (GFP^+^) and non-effectively transduced (GFP^−^) cells.

**Figure 5:**
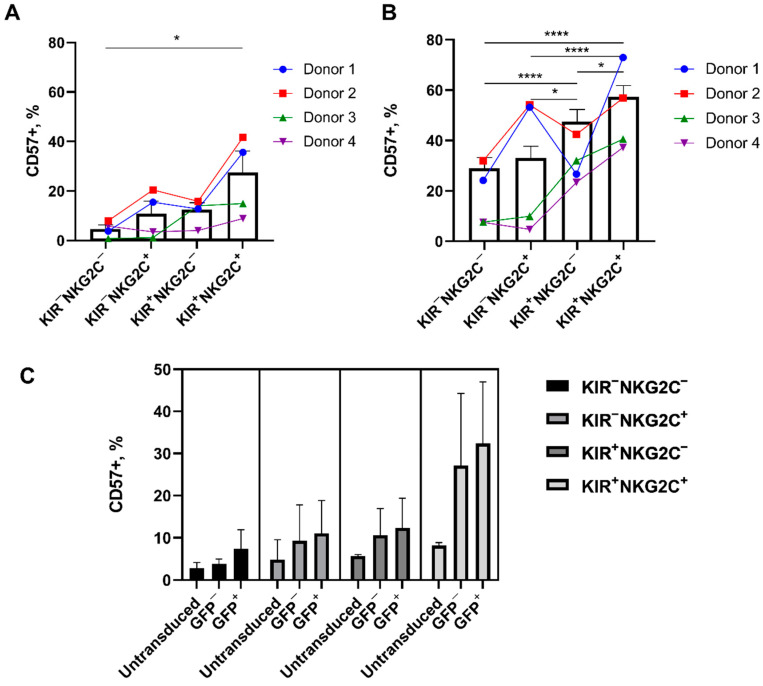
The proportion of CD57^+^ NK cells ex vivo—in the cultures obtained after stimulation with K562-mbIL21 and IL-2 before the transduction and in the cultures of the transduced cells. (**A**) The proportion of CD57^+^ NK cells in cultures derived from CD57-negative subpopulations differing in the expression of KIR2DL2/3 and NKG2C after 7 days of stimulation with K562-mbIL21 and IL-2; mean ± SE and individual values of 4 donors selected for transduction experiments are shown. (**B**) The proportion of CD57^+^ cells ex vivo in NK subpopulations differing in the expression of KIR2DL2/3 and NKG2C from 17 healthy donors (bars, averaged), and from the 4 donors selected for transduction experiments (colored dots and lines). (**C**) The proportion of CD57^+^ cells in the cultures of untransduced, non-effectively transduced (GFP−), and effectively transduced (GFP+) NK cells from the subpopulations that initially did not express CD57 and differed in the expression of NKG2C and KIR2DL2/3, 7 days after the transduction, *n* = 3. (**A**–**C**) Statistical analysis was performed using a paired *t*-test (* *p* < 0.05; **** *p* < 0.0001). Columns present means ± SE. Data of the individual donors are shown by the lines.

NK cells started expressing CD57 de novo in all the studied subpopulations after stimulation for seven days with K562-mbIL21 and IL-2. The largest proportion of newly obtained CD57^+^ cells was observed in the highly differentiated CD57^−^KIR2DL2/3^+^NKG2C^+^ subset, while the smallest was in the less differentiated CD57^−^KIR2DL2/3^−^NKG2C^−^ subset (Figure 5A). The level of CD57-positive cells in these cultures correlated with the initial proportion of CD57^+^ cells in the ex vivo KIR2DL2/3^+/−^NKG2C^+/−^ subpopulations (Figure 5B). The proportion of CD57^+^ cells was initially the smallest, and the de novo expression of CD57 appeared at a lesser extent in the least differentiated KIR2DL2/3^−^NKG2C^−^ subpopulation compared to other subsets. Similarly, in the most differentiated KIR2DL2/3^+^NKG2C^+^ subset, the proportion of CD57^+^ cells was initially higher ex vivo (Figure 5B).

A higher proportion of CD57^+^ cells was observed in both GFP^−^ and GFP^+^ transduced NK cells compared to untransduced cells in all the subsets except for KIR2DL2/3^−^NKG2C^−^ (Figure 5C). In the CD57^−^KIR2DL2/3^+^NKG2C^+^ subset, the proportion of CD57^+^ cells increased to the greatest extent in all transduced NK cells relative to untransduced cells.

**Figure 6:**
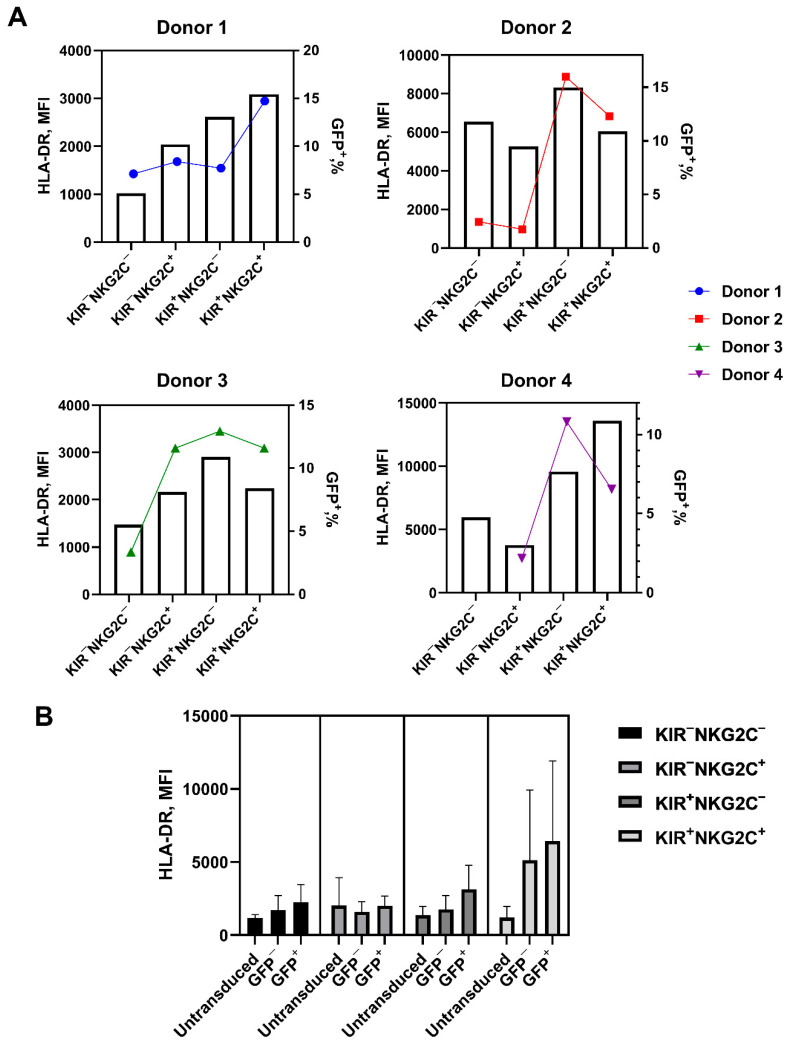
HLA-DR expression level in the cultures of transduced and untransduced NK cells from the subpopulations that initially did not express CD57 and differed in the expression of NKG2C and KIR2DL2/3. (**A**) HLA-DR expression level in the cultures of the studied subsets from the four individual donors after 7 days of stimulation with IL-2 and feeder cells. The left Y axis is HLA-DR expression level (bars). The right Y axis is the transduction level (colored dots and lines). (**B**) HLA-DR surface expression level in the cultures of the transduced and untransduced NK cells from the studied subpopulations on day 7, after the transduction.

As we have shown earlier [25], NK cell stimulation by a combination of IL-2 and K562-mbIL21 feeder cells promotes the expression of the HLA-DR activation marker on the cell surface. In this work, more than 90% of the NK cells in all subpopulations expressed HLA-DR on their surface after stimulation with IL-2 and K562-mbIL21 for 7 days. In the majority of the cases, an association between HLA-DR expression level and the proportion of GFP-expressing cells after the transduction was observed; the highest level of HLA-DR expression corresponded with the greater susceptibility to transduction (Figure 6A).

Additionally, the density of HLA-DR surface expression was slightly increased on the surface of GFP^+^ cells compared to untransduced and GFP^−^ cells. For the KIR2DL2/3^+^ subpopulations, the greatest increase in the expression level of this activation marker was observed (Figure 6B).

### 2.6. Verification of iCasp9 Transgene Stability in Modified NK Cells by Monitoring Reporter Gene Expression Level

**Figure 7:**
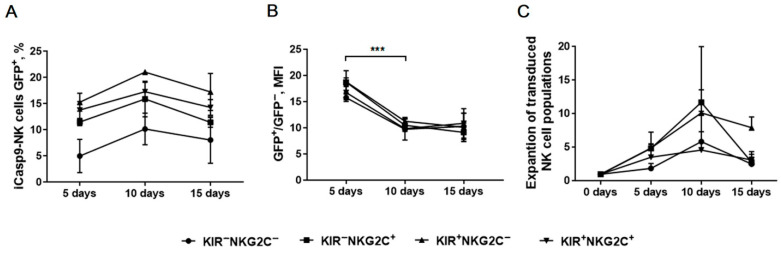
The dynamics of the GFP^+^ iCasp9-NK cells, *n* = 2 (donors 2 and 3). (**A**) The dynamics of the proportion of the GFP^+^ iCasp9-NK cells in the indicated subsets. (**B**) A comparison of GFP mean fluorescence intensity (MFI) normalized on the MFI of the GFP^−^ NK cells among the indicated NK cell populations during long-term cultivation. (**C**) A comparison of the iCasp9-NK cell number after the transduction, normalized on the initial number of transduced cells. (**A**–**C**) Statistical analysis was performed using a paired *t*-test (*** *p* < 0.001); means ± SE are shown.

We next traced the dynamics of iCasp9-NK cells for 10 days in each subset culture containing both GFP^−^ and GFP^+^ cells after the transduction in order to check for transgene stability through the registration of GFP fluorescence. Statistical difference in the level of the reporter protein expression was not detected between the subsets (Figure 7A). The level of GFP fluorescence intensity dropped similarly with time in all the NK cell populations studied (Figure 7B), along with the growing proliferative activity of these cells (Figure 7C). The obtained results indicate no proliferation advantage between GFP^+^ and GFP^−^ NK cells. A decrease in the level of transgene expression may be associated with its down-regulation after retroviral transduction as it was previously shown for mammalian cells [26]. However, we cannot unambiguously exclude the role of unintended spontaneous dimerization of the iCasp9 suicide construction due to its high concentration in the cells, which led to cell death.

### 2.7. The Response of iCasp9-NK Cells to the Chemical Inductor of Dimerization in the CD57^−^KIR2DL2/3^+/−^NKG2C^+/−^Subsets

**Figure 8:**
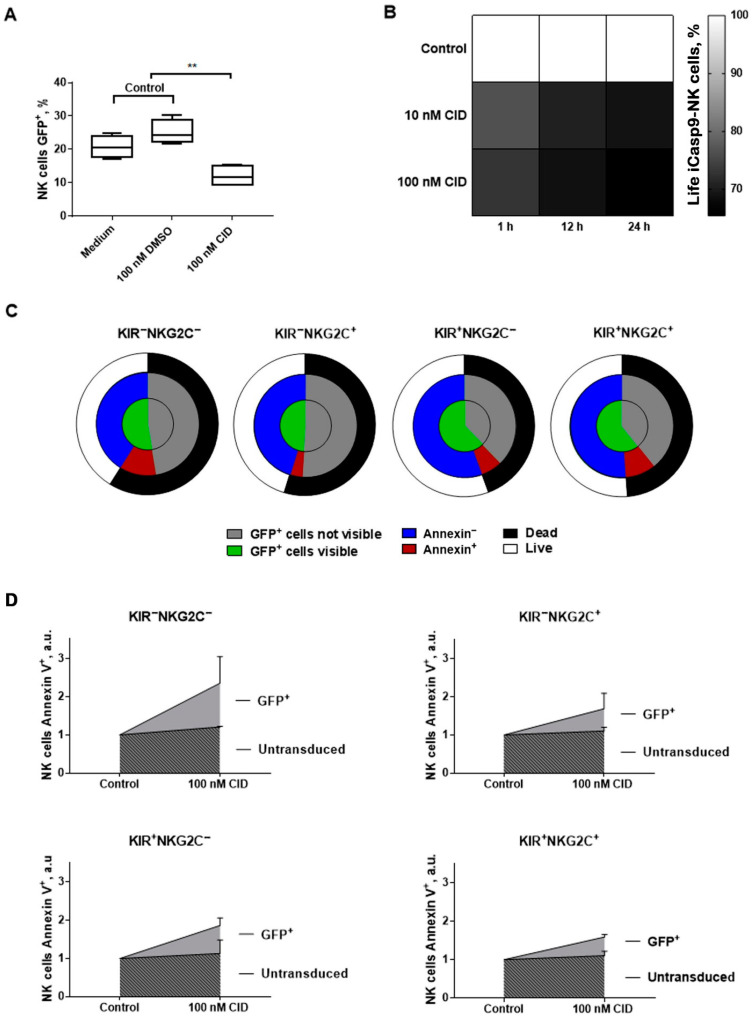
Apoptosis induction in iCasp9-GFP-positive NK cells by chemical inductor of dimerization (CID) (**A**) The proportion of live GFP^+^ NK cells after a 24 h incubation with the medium, 100 nM DMSO and 100 nM CID, *n*=3. (**B**) A comparison of live iCasp9-NK cells after incubation for 1 h, 12 h, and 24 h, with 10 nM and 100 nM CID. The percentage of live cells after incubation with CID normalized to the fraction of live iCasp9-NK cells in controls (medium and 100 nM DMSO) is shown. The mean percentage is presented (*n* = 2). (**C**) Proportions of life/dead iCasp9-NK cells after a 24 h incubation with 100 nM CID normalized to the total fractions of life/dead cells in the controls, *n* = 2. The fraction of dead iCasp9-NK cell (black) consists of Annexin V^+^ and/or SytoxVioBlue^+^ cells (red), and cells destroyed before measurement and not visible by flow cytometry as GFP^+^ NK cells (gray). The fraction of live cells (white) consists of GFP^+^ NK cells (green) and Annexin V^−^ SytoxVioBlue^−^ NK cells (blue). (**D**) Proportions of Annexin V^+^ GFP^+^ cells in arbitrary units (a.u.) for the KIR2DL2/3^−^NKG2C^−^, KIR2DL2/3^−^NKG2C^+^, KIR2DL2/3^+^NKG2C^−^, and KIR2DL2/3^+^NKG2C^+^ NK cell subsets after incubation with the medium or 100 nM CID, *n* = 2 (donors 2 and 3). Statistical analysis was performed using a paired *t*-test (** *p* < 0.01); means ± SE are shown.

Next, we analyzed the incorporation and functionality of the iCasp9 suicide gene in GFP^+^ NK cells (Figure 8). First, we verified that the presence of DMSO (CID solvent) in the medium did not affect cell survival (Figure 8A). The addition of CID resulted in a decrease in the GFP^+^ cell fraction in the transduced NK cells (Figure 8A). CID did not increase the level of apoptosis in non-modified NK cells, whereas a noticeable proapoptotic effect of CID was observed in GFP+ iCasp9-NK cells (Figure 8D). CID concentration and duration of incubation influenced the proportion of live iCasp9-NK cells, with the highest percentage of dead cells observed for iCasp9-NK cells incubated with 100 nM CID for 24 h (Figure 8B). 

The incubation of iCasp9-transduced KIR2DL2/3^−^NKG2C^−^, KIR2DL2/3^−^NKG2C^+^, KIR2DL2/3^+^NKG2C^−^, and KIR2DL2/3^+^NKG2C^+^ NK cell subsets for 24 h with 100 nM CID led to a decrease in the proportion of GFP^+^ NK cells visible by flow cytometry, possibly as a result of the decay of dead cells (Figure 8C and Figure S2). The fraction of live iCasp9-NK cells decreased, and the proportion of iCasp9-NK cells in apoptosis detected by Annexin V and SytoxVioBlue staining increased (Figure 8C and Figure S2). The increase in the MFI level of Annexin staining was also demonstrated for iCasp9-NK cells incubated with CID (Figure S2).

It is worth mentioning that NK cells modified with the iCasp9 gene were incubated with CID on day 11, after the transduction. At this time period, the transduced NK cells demonstrated high proliferative activity (Figure 7C). We revealed higher CID-mediated apoptosis induction responses in the KIR2DL2/3^−^NKG2C^−^ and KIR2DL2/3^−^NKG2C^+^ iCasp9-subsets as compared to the KIR2DL2/3^+^NKG2C^−^ ang KIR2DL2/3^+^NKG2C^+^ iCasp9-subsets. Nevertheless, there were some GFP^+^ iCasp9-NK cells that still remained alive after incubation with CID. Difference between donors was observed in the death induction response upon CID treatment of the iCasp9-NK cells. We also found that more differentiated NK cells (according to CD57 and KIR2DL2/3 expression) appeared to be more resistant to the induction of CID-mediated apoptosis (Figure 5A and Figure 8C,D). To sum up, various factors, including the NK cell differentiation state, proliferative rate, and activation status along with the iCasp9-transgene expression level, may affect the regulation of the life/death signaling balance of iCasp9-transduced NK cell populations.

## 3. Discussion

The application of NK cells against malignant neoplasms is a developing and prospective area in immunotherapy. NK cells have several advantages over T cells [27]. There are also certain benefits in using adaptive-like NK cells in immunotherapy: NKG2C^+^ NK cells are associated with preventing GVHD [28] and demonstrate higher ADCC levels [29,30]; they are also capable of not only destroying malignant HLA-Epos neoplasms, but also have increased allo-reactivity in HLA-incompatible recipients due to the shift of the KIR repertoire towards self-KIRs [15]. The NKG2C^+^CD57^+^ NK cell subset often occurs among people infected with HCMV. This infection induces the expansion and differentiation of KIR-expressing NK cells [15]. The KIR^+^CD57^+^NKG2C^+^ NK cell subset is characterized by a “memory-like” phenotype [31], but because of the low proliferative potential, the genetic modification of these cells remains a problem. At the same time, some of the NKG2C^+^CD57^−^ NK cells can exhibit adaptive properties while demonstrating a better proliferative potential [17]. Therefore, while bearing in mind that the genetic modification of CD57^+^ NK cells is not efficient [6], in this work, we decided to investigate the effectiveness of the retroviral transduction of less differentiated CD57^−^ for NK cell subsets which precede “memory-like” NK cells. In particular, the retroviral transduction of the NKG2C^+^CD57^−^ NK cell subset may help to overcome problems with the modification of less susceptible NKG2C^+^CD57^+^ NK cells. 

The acquisition of the KIR surface expression by NK cells is linked to the process called “licensing”. During this maturation stage, NK cells modulate their activation response and increase functional activity [32]. To study the differences between the KIR^−^ and KIR^+^ stages of NK cell maturation, we divided CD57^−^ NK cells into subsets based on the expression of the most common inhibitory KIRs—KIR2DL2 and KIR2DL3. Hence, we studied the following CD56^+^CD57^−^NK cell subsets: KIR2DL2/3^−^NKG2C^−^, KIR2DL2/3^−^NKG2C^+^, KIR2DL2/3^+^NKG2C^−^, and KIR2DL2/3^+^NKG2C^+^.

We have analyzed the transduction efficiency of NK cells pre-stimulated with K562-mbIL12 feeder cells and IL-2 and their relationship with their proliferative potential, expansion rate, survival, and phenotype. We revealed a higher proportion of GFP^+^ iCasp9-NK-cells in the KIR2DL2/3^+^ subpopulations in comparison with the KIR2DL2/3^−^ subsets, while the level of GFP fluorescence intensity was significantly higher in the KIR2DL2/3^−^NKG2C^+^ subset compared to the KIR2DL2/3^−^NKG2C^−^ subset in each experiment (Figure 3). To figure out the features that could cause the observed differences in NK cell susceptibility to retroviral transduction, we investigated the phenotype and proliferative activity of the CD57^−^KIR2DL2/3^+/−^NKG2C^+/−^ NK cell subsets. As we have shown earlier, NK cells stimulated with IL-2 and K562-mbIL21 feeder cells increase the surface expression of the HLA-DR activation marker [26]. Here, we revealed that the increase in HLA-DR expression is associated with a larger fraction of GFP^+^ cells in all NK cell subsets. Especially high HLA-DR surface expression density was reported for the CD57^−^KIR2DL2/3^+^ subsets, which were in turn characterized by a higher fraction of GFP^+^ cells (Figure 6A).

We have also shown that CD57 expression appears de novo in the studied NK cell subpopulations during cultivation after the transduction. The CD57 expression increase is the most prominent in the most differentiated CD57^−^KIR2DL2/3^+^NKG2C^+^ subset and less prominent in the least differentiated KIR2DL2/3^−^NKG2C^−^ subset. These data are consistent with the results we obtained earlier [8]. It was discussed above that CD57^−^NKG2C^+^ NK cells can be precursors of adaptive cells, which retain the increased proliferative potential while acquiring CD57 surface expression. It is possible that the appearance of the CD57 expression that we observed is associated with the transition of CD57^−^NKG2C^+^ NK cells to a more adaptive-like state.

Expansion and proliferation rates were examined for all studied subpopulations, since according to our previous data [23], the highest transduction efficiency was observed among actively proliferating NK cells. The main NK cell cultures in our research remained heterogeneous despite preliminary sorting, so the contribution of each individual cell to the culture expansion was unknown. In order to elucidate this issue we cloned NK cells from each studied subset using the “single cell” mode of the cell sorter. We have investigated patterns related to the clone formation efficiency, the number of cells formed in each clone, and the contribution of the CD56^bright^ and CD56^dim^ NK cell subpopulations. As shown in Figure 4, the CD57^−^KIR2DL2/3^+^ subsets characterized by a higher proportion of more differentiated CD56^dim^NK cells produced smaller numbers of abundantly proliferating clones. It can be suggested that in the KIR2DL2/3^+^ NK cell fraction, only a small number of actively proliferating clones can be transduced, and these transduced clones will continue proliferating in parallel with unmodified cells. Whereas in the case of the KIR2DL2/3^−^ NK cell fraction, the proliferative response to the IL-2/K562-mbIL21 stimulation happens mostly due to the expansion of multiple individual cells, and the contribution of genetically modified NK cells becomes less prominent. The cell numbers in the CD56^bright^ KIR2DL2/3^−^ NK cell cultures decreased from day 14 to day 18, while the cell numbers in the KIR2DL2/3+ NK cell cultures increased (Figure 4D). Thus, a higher transduction level in KIR2DL2/3^+^ cells is consistent with these observations as long as CD56^bright^KIR2DL2/3^+^ cells showed a higher proliferative response during activation by IL-2 and feeder cells.

Constructs containing the suicide gene are now used to control long-lived genetically modified lymphocytes. The application of inducible caspase 9 (iCasp9), which is activated by binding to a chemical inducer of dimerization (CID) [20], has proven its effectiveness in the elimination of CAR-NK cells in both in vitro and in vivo preclinical studies [33]. However, several problems still potentially exist for the iCasp9 suicide system. For example, the loss of transgene expression that is consequently often observed, due to the down-regulation of the iCasp9 gene, after the retroviral transduction of mammalian cells [27]. In this work, we used the pMSCV-F-del Casp9.IRES.GFP construct. This transgene was flanked by retroviral integrants with the chicken beta-globin chromatin insulator [34]. Previously, it was shown that this modification drastically uniformed the transgene expression in transduced 293T cells, but did not significantly influence the transduction of primary T cells [35]. We did not detect any decrease in the proportion of modified (GFP^+^) cells throughout the experiments (Figure 7A). However, a spontaneous dimerization of iCasp9 may be an additional problem for this system, which can lead to a decrease in proliferative potential and undesirable cell death. Such effect was observed in inducible systems based on Fas [36,37]. For iCasp9-modified cells, a low level of spontaneous mortality and long-term persistence in vivo was described earlier [38], which corresponds to our data (Figure 7A,C). However, a drop of GFP fluorescence was observed in all NK subsets studied (Figure 7B). We cannot simply point out the causes of this phenomenon, but we do not exclude the possibility of spontaneous iCasp9 dimerization, especially in conditions of its possible rapid accumulation as a consequence of active proliferation in the first days after the transduction.

The resistance of modified cells to cell death induction upon treatment with CID is another problem for the iCasp9-based suicide system. The upregulation of anti-apoptotic genes results in the enhanced survival of the modified cells. Such interruption of cell death induction in cells modified with suicide-gene-bearing constructions has already been observed in other suicide systems that involved various elements of programmed cell death pathways, including the Fas-mediated pathway [39]. In comparison with other proteins of the apoptotic cascade, the activation of caspase 9 occurs at the later stages of the apoptotic pathway and is therefore expected to avoid inhibition by anti-apoptotic regulators such as c-FLIP and BCL-2 family members [35].

We found that a fraction (about 20%) of iCasp9-NK cells with no signs of apoptosis induction still remains after a 24 h incubation with 100 nM of CID. It was shown in an earlier work that a 30 min incubation with a 10 nM CID was sufficient to activate iCasp9 in all iCasp9-NK-92 cells, and the proportion of living iCasp9-NK-92 cells decreased up to 2% after a 26 h incubation with CID [21]. Incomplete elimination was described for both iCasp9-modified cell lines such as Karpas 299 [40] and iCasp9-HSPC, and was probably associated with the increased expression of anti-apoptotic factors, particularly Bcl2 [40]. Despite the fact that Bcl2 lies upstream in the mitochondria death triggering pathway than in the caspase 9 and iCasp9 construct, Bcl2 indirectly affects the activity of caspase inhibitors such as IAPs (inhibitors of apoptosis proteins) [41]. Furthermore, NK cells that have passed through the “license” stage of differentiation obtain a higher resistance to cell death due to increased expression in the anti-apoptotic proteins of the BCL2 family (Bcl2 and Bcl-_XL_). Improved survival was shown for CD57^+^ NK cells expressing inhibitory KIRs and NKG2A/C as compared to the cells lacking these surface molecules [42]. In addition, the expression of the BCL2-member proteins that increases under signaling from the common γ-chain of IL-receptors (IL-2R, IL-15R. IL-21R) mediates the differentiation and survival of more mature NK cells [43]. Thus, we assume that an increased survival rate of iCasp9-NK cells in the KIR^+^NKG2C^+^ subset may be linked to an increased level of Bcl2 expression in these cells, which in turn corresponds to a higher CD57 surface expression on the transduced cells. While CD57^+^ NK cells are characterized as more differentiated cells with low proliferative potential [11], that also indicates a possible connection between the enhanced death resistance and Bcl2 accumulation among more differentiated resting NK cells [43]. On the other hand, low iCasp9 transgene expression level may contribute to incomplete apoptosis induction in cells upon CID treatment. However, it was reported that cells, after sorting by the reporter gene expression, increased their susceptibility to CID by up to 99% [35].

## 4. Materials and Methods

### 4.1. Cell Lines

Genetically modified cells with membrane, IL-21 K562 (K562-mbIL21), were kindly provided by Dr. Dean Lee (MD Anderson Cancer Center, Houston, TX, USA). These cells also expressed CD64, CD86, CD137L, and the fragment of the CD19 receptor [7]. The erythroblastic leukemia cell line K562 was acquired from ATCC (Manassass, VA, USA). The K562 and K562-mbIL21 cell lines were cultivated in an RPMI-1640 medium (PanEco, Moscow, Russia) supplemented with 10% fetal calf serum (FCS, HyClone Labs, Logan, UT, USA), 2 mM L-glutamine (PanEco, Moscow, Russia), and 2 mM antibiotic-antimycotic. The cells γ-irradiated at 100 Gy K562-mbIL21 were applied as a feeder for NK cell stimulation. The NK-92 cell line was obtained from ATCC (Manassass, VA, USA). This cell line was cultivated in an RPMI-1640 medium (PanEco, Moscow, Russia), supplemented with 10% fetal calf serum (FCS, HyClone Labs, Logan, UT, USA), 2 mM L-glutamine (PanEco, Moscow, Russia), 2 mM sodium pyruvate (PanEco, Moscow, Russia), 4 mM of non-essential amino acids (PanEco, Moscow, Russia), and 2 mM antibiotic-antimycotic (Sigma-Aldrich, St. Louis, MO, USA), with an addition of 200 U/mL of IL-2 (Hoffmann La-Roche, Basel, Switzerland) and 5γ/mL of IL-15 (Sigma-Aldrich, St. Louis, MO, USA). The Phoenix Ampho cell line constructed on the basis of the HEK293T pancreatic cell line was used for retroviral packaging due to its constitutive expression of viral genes: *Gag-Pol/Tat/Env/Rev*. These cells were kindly provided by Dr. Alexander Filatov (SSC “Institute of Immunology”, Moscow, Russia). Phoenix Ampho cells were cultured in a DMEM medium (PanEco, Moscow, Russia), supplemented with 10% FCS, 2 mM L-glutamine, 2 mM sodium pyruvate (PanEco, Moscow, Russia), 2 mM antibiotic-antimycotic (Sigma-Aldrich, St. Louis, MO, USA).

### 4.2. NK Cell Isolation

Blood samples were taken from healthy donors, who gave their informed consent (approved by the local ethics committee of the Pirogov Russian National Medical University). The fraction of peripheral mononuclear blood cells (PBMC) was obtained by gradient centrifugation in a standard Ficoll solution with a density of 1.077 (PanEco, Moscow, Russia). NK cells were isolated by negative magnetic separation from PBMC using the NK cell isolation kit (MiltenyiBiotec, Bergisch Gladbach, Germany) in accordance with the manufacturer’s protocol.

### 4.3. HCMV Serology Status

Along with blood samples, serum samples were collected from each donor. The HCMV status was determined by measuring HCMV-specific IgG titer in sera samples using ELISA kit (Vector-Best, Novosibirsk, Russia), following the manufacturer’s protocol.

### 4.4. Generation of NK Cell Clones and Populations

The FACSVantageDiVa cell sorter (Becton Dickinson, Franklin Lakes, NJ, USA), equipped with 405, 488, and 643 nm lasers and a corresponding set of detectors and filters, was used for cell sorting. Prior to sorting, freshly isolated NK cells were placed in the separation buffer (PBS containing 0.5% of BSA and 2 mM EDTA) and then labeled with mouse anti-human antibodies (Abs) specific for CD56, NKG2C, KIR2DL2/3, CD57 for the isolation of the CD57^−^KIR2DL2/3^−^ and CD57^−^KIR2DL2/3^+^ subsets, or the CD57^−^KIR2DL2/3^−^NKG2C^−^, CD57^−^KIR2DL2/3^−^NKG2C^+^, CD57^−^KIR2DL2/3^+^NKG2C^−^, and CD57^−^KIR2DL2/3^+^NKG2C^+^ subsets. In the first case, sorted cells were placed in 24-well plates in a concentration 700,000–900,000 cells per well and then cultivated in an NK MACS cell medium (MiltenyiBiotec, Bergisch Gladbach, Germany). In the latter case, the cells were divided into three parts and then sorted and cultured in different conditions. The first portion of NK cells was sorted into four collection tubes, each for one of the indicated subsets. Gating strategy and the sorted subset purity data are presented in Figure S1. Sorted cells were placed in 96-well flat-bottom plates at a concentration of 25,000 cells per well and then cultivated in the medium for clones (20% ExVivo medium (ThermoFisher Scientific, Carlsbad, CA, USA), 80% DMEM medium (PanEco, Moscow, Russia), 2 mM L-glutamine, 2 mM sodium pyruvate (PanEco, Moscow, Russia), 2 mM antibiotic-antimycotic (Sigma-Aldrich, St. Louis, MO, USA)) with 10^5^ K562-mbIL21 feeder cells per ml and 100 U/mL of recombinant IL-2 (Sigma-Aldrich, St. Louis, MO, USA). Another portion of these cells was sorted into wells of a 96-well U-bottom plate, 100 cells per well. The indicated subsets (CD57^−^KIR2DL2/3^−^NKG2C^−^, CD57^−^KIR2DL2/3^−^NKG2C^+^, CD57^−^KIR2DL2/3^+^NKG2C^−^, CD57^−^KIR2DL2/3^+^NKG2C^+^) were sorted from the CD56^bright^ NK cell subpopulation. These cells were cultivated in the medium for clones, with 10^4^ K562-mbIL21 feeder cells per ml and 100 U/mL of recombinant IL-2 (37 °C, 5% CO_2_). Half of the culture medium in the wells was replaced thrice a week. Cells mostly grew at a density of 50,000–100,000 per well in 96-well plates. When the total size of each subset exceeded 700,000 cells, they were placed into wells of 24-well plates where they grew at a density of 700,000–900,000 cells per well. The third portion of freshly isolated and Abs-stained NK cells was sorted in the “single cell” mode into 96-well U-bottom plates, one cell per well, and then cultivated for 5 weeks (37 °C, 5% CO_2_) in the medium for clones, with K562-mbIL21 feeder cells (10^4^ cells per mL) and 100 U/mL of recombinant IL-2 (Sigma-Aldrich, St. Louis, MO, USA). The subpopulations CD57^−^KIR2DL2/3^−^NKG2C^−^, CD57^−^KIR2DL2/3^−^NKG2C^+^, CD57^−^KIR2DL2/3^+^NKG2C^−^, CD57^−^KIR2DL2/3^+^NKG2C^+^ were cloned. In three weeks, half of the medium was replaced with a fresh medium. The clones were counted by week 5.

### 4.5. Cell Staining and Flow Cytometry

MACSQuant 10 cytometer (Miltenyi Biotech, Bergisch Gladbach, Germany) equipped with 405 nm, 488 nm, and 635 nm lasers was used for the acquisition of flow cytometry data. The following mouse anti-human Abs were used for extracellular staining: CD56-BrilliantViolet (clone HCD56), CD56-APC (clone HCD56), CD107a-APC (clone H4A3), HLA-DR-BrilliantViolet 421 (clone L243) (Sony Biotechnology, San Jose, CA, USA), CD56-APC-Vio770 (clone REA196), CD57-VioBlue (clone TB03), CD57-PE-Vio770 (clone REA769), KIR2DL2/3-PE (clone DX27), KIR2DL2/3-PE-Vio615 (clone REA1006) (Miltenyi Biotech, Bergisch Gladbach, Germany), NKG2C-AlexaFluor (AF) 488 (clone 108724), NKG2C-PE (clone 134591) (R&D Systems, Minneapolis, MN, USA), HLA-DR-PE-Cy7 (clone Immu357) (Beckman Coulter, Miami, FL, USA). Abs-stained cells were incubated for 30 min at 4 °C in PBA staining buffer (PBS containing 0.5% BSA (Serva, Heidelberg, Germany) and 0.01% sodium azide (AMRESCO Inc., Aurora, CO, USA)), then washed with PBS. No less than 30,000 events for NK cells in an FSC/SSC gate and no less than 5000 events for NK cell fractions were recorded. Phenotype of NK cells had been measured on the 7th day of culturing for non-transduced cells and on the 7th day after transduction for both untransduced and transduced cells.

### 4.6. Proliferative Capacity Measuring

NK cells from each culture of each subset were counted on days 6, 10, and 11 after sorting using cell counter TC20 (Bio-Rad Laboratories, Hercules, CA, USA). Transduced cells were counted on days 4 and 10 after the transduction. Proliferative capacity was determined by the expansion coefficient, calculated using the following formula: K = number of cells on day X/number of cells on day 0.

### 4.7. Retroviral Transduction

The iCasp9 gene was delivered to stimulated NK cells with the use of pMSCV-F-del Casp9.IRES.GFP plasmid (Addgene #15567) or test GFP^+^ plasmid (xlox-GFP-hTERT, Addgene #69809). The vector was pseudotyped with RD114 envelope glycoprotein to enhance transduction efficiency [44]. The plasmid purification was conducted with Plasmid Midiprep 2.0 kit (Evrogen, Moscow, Russia) according to the manufacturer’s instructions. The plasmid concentration was measured by Biodrop (Innovative Solutions, Carson, NV, USA). For all experiments performed, only one unified viral stock was used. To acquire high viral titer, Phoenix Ampho cells were transfected using a calcium phosphate transfection kit (ThermoFisher Scientific, San Jose, CA, USA) according to the manufacturer’s instructions. A total of 150 mm Petri dishes were treated with poly-L-lysine solution (Sigma-Aldrich, St. Louis, MO, USA). Plasmids carrying iCasp9 and RD114 were co-transfected at a ratio of 2:1 (144 µg iCasp9 plasmid + 72 µg RD114 plasmid per dish). Viral supernatant was harvested and filtered through Millex-HV-0.45 µm PES filter (Millipore, Burlington, MA, USA) at 24, 48, 72, and 96 h post-transfection. Aliquots were concentrated by centrifugation at 21,000× *g*, 4 °C, 2.5 h. The retroviral transduction of NK92 and stimulated NK cell populations was performed in 96-well plates coated with 20 μg/mL Retronectin solution (Clontech/Takara, Terra Bella Ave. Mountain View, CA, USA) in PBS, according to the manufacturer’s recommendations. The transduction efficiency was determined by flow cytometry according to the expression of the green fluorescent protein (GFP). The number of transduction units was calculated using the formula: Titer = TU/mL (number of transduction units per ml = number of transduced cells * fluorescence level, %/viral volume, mL/100%).

### 4.8. Cell Death Induction in iCasp9-NK Cells by Chemical Inductor of Dimerization

The iCasp9-NK cells along with unmodified NK cells were incubated for 24 h in a medium, with the addition of: PBS, DMSO, and 100 nM of chemical inductor of dimerization (CID) (AP20187 (MedChemExpress, Monmouth Junction, NJ, USA). DMSO was taken at a concentration corresponding to its content in a sample with 100 nM CID. The level of apoptosis and cell viability were measured by flow cytometry with the use of AnnexinV-AF647 (Invitrogen, San Jose, CA, USA) and Sytox-VioBlue (Invitrogen, San Jose, CA, USA).

### 4.9. Data Analysis

The results were processed using the FlowJo software version X (TreeStar Williamson Way, Ashland, OR, USA). Statistical analysis was performed using the GraphPad Prism 7 software (StatSoft Inc., Tulsa, OK, USA). Paired Student’s *t*-test was applied for data that passed the Shapiro–Wilk normality test and the Mann–Whitney test for data not normally distributed. Means ± SEM are presented throughout the paper (* *p* < 0.05; ** *p* < 0.01; *** *p* < 0.001; **** *p* < 0.0001).

## 5. Conclusions

We investigated the relationship between different functional characteristics of NK cells and their susceptibility to retroviral transduction with the iCasp9 gene, followed by the CID-mediated elimination of modified cells. We showed that subpopulations of activated NK cells expressing KIR and NKG2C on their surface have the highest iCasp9 transduction levels, which was also associated with the increased proliferative potential of these subpopulations at later stages of cultivation. In addition, there was an association between a high level of the activation marker HLA-DR, the expression of de novo CD57, and a higher proportion of transduced cells in the subsets. The sensitivity of iCasp9-transduced cells to the induction of apoptosis by CID was also examined. The NK cells that expressed KIR2DL2/3 had the weakest responses to the induction of CID-mediated apoptosis. Knowledge about the iCasp9 retroviral transduction efficiency in various subpopulations of NK cells and their subsequent response to apoptosis induction by CID is important in the context of NK cell usage in immunotherapy.

## Supplementary Materials

The following are available online at https://www.mdpi.com/article/10.3390/ijms222413326/s1.
