# Supplementary figures and images for: Increased Susceptibility of the CD57− NK Cells Expressing KIR2DL2/3 and NKG2C to iCasp9 Gene Retroviral Transduction and the Relationships with Proliferative Potential, Activation Degree, and Death Induction Response
Source: Int J Mol Sci. 2021 Dec 11;22(24):13326. doi: 10.3390/ijms222413326 (PMC8709225; doi:10.3390/ijms222413326)

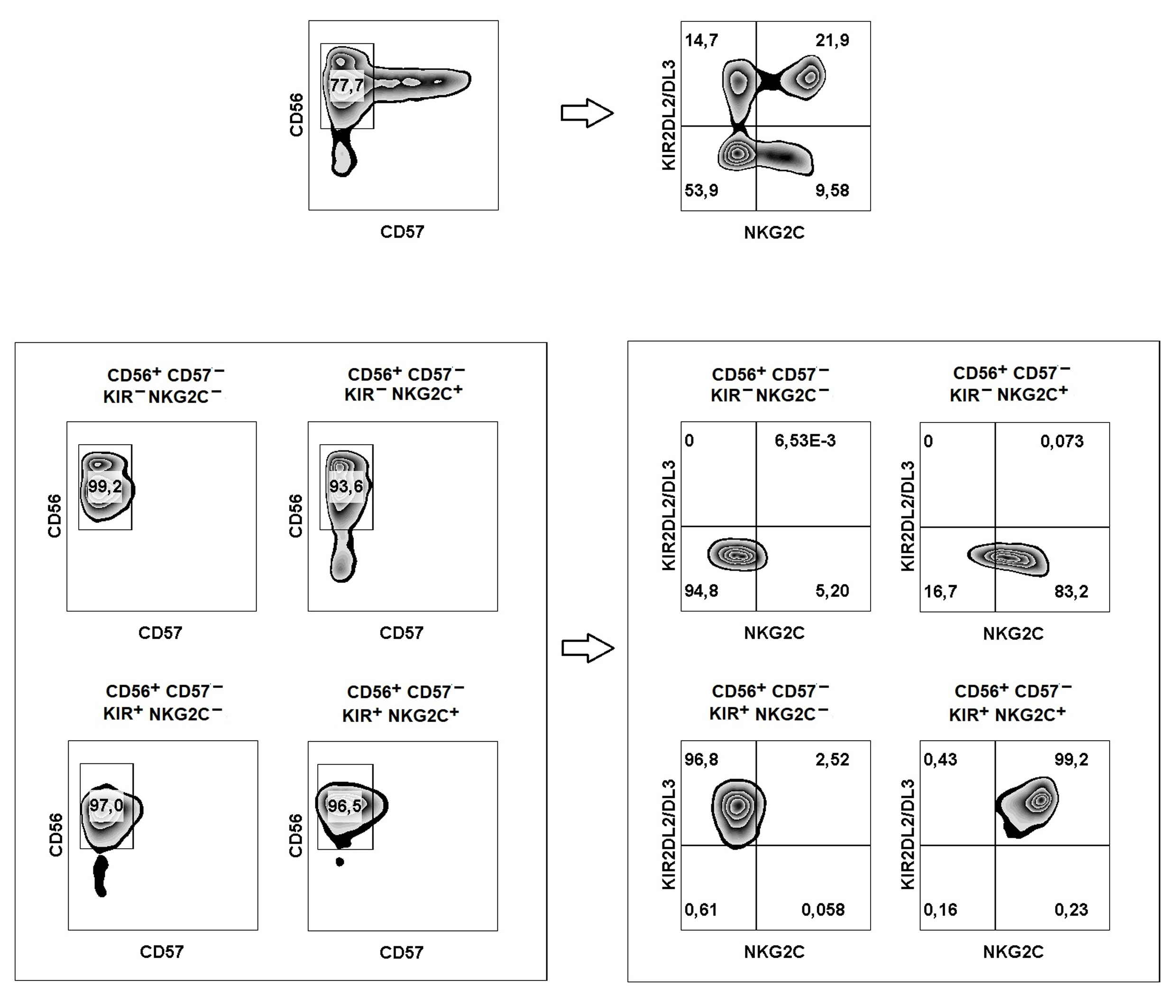

Supplement: Supplementary file 1 [file ijms-22-13326-s001.zip › Supplementary Figure 1.tif]

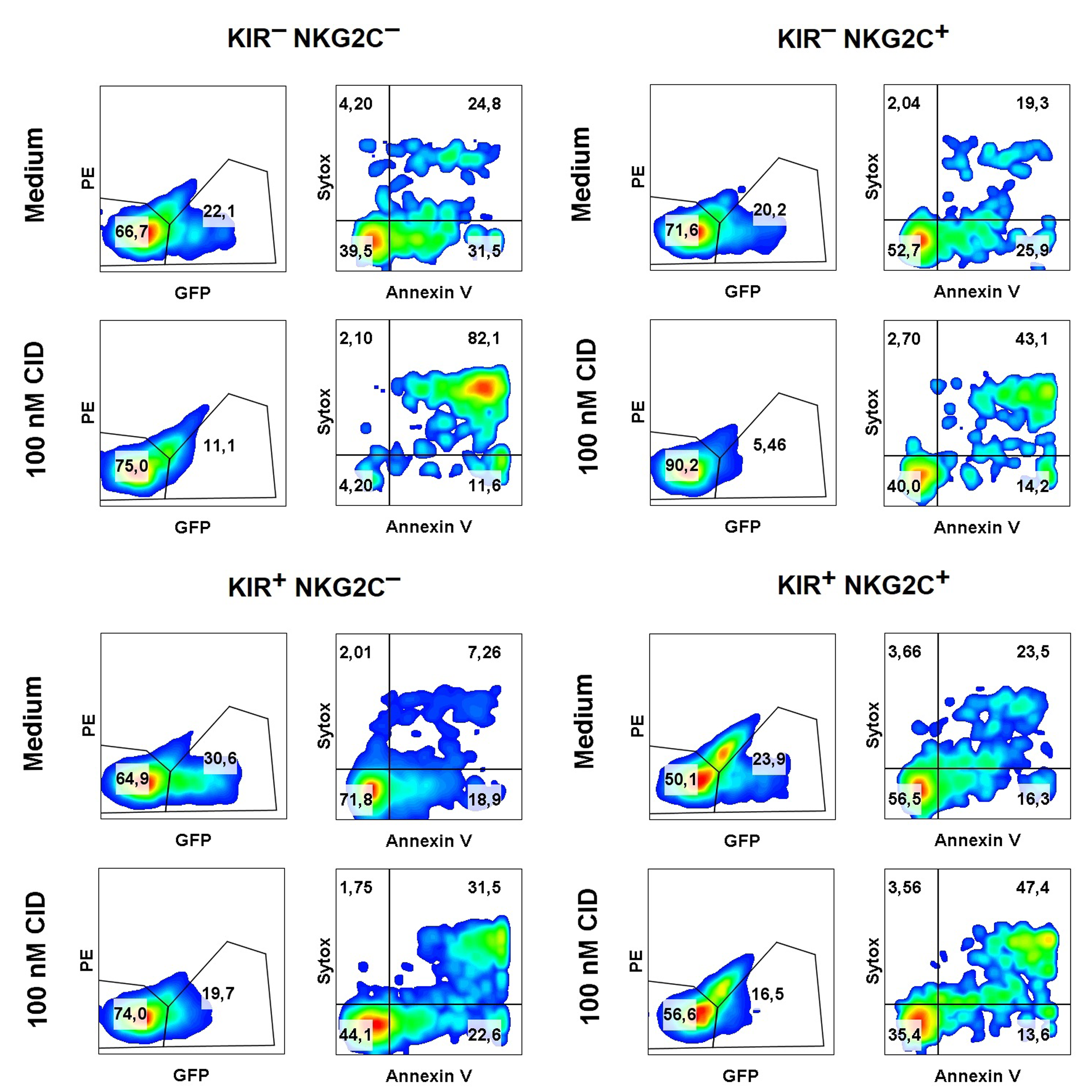

Supplement: Supplementary file 1 [file ijms-22-13326-s001.zip › Supplementary Figure 2.tif]
